# Shared decision making for women with uncomplicated Cystitis in Primary Care in the Netherlands: a qualitative interview study

**DOI:** 10.1186/s12875-022-01867-9

**Published:** 2022-10-05

**Authors:** Tessa M.Z.X.K. van Horrik, Bart J. Laan, Rosanne van Seben, Gerda Rodenburg, Edwin J. Heeregrave, Suzanne E. Geerlings

**Affiliations:** 1 Department of Internal Medicine, Amsterdam UMC location University of Amsterdam, Infectious Diseases, Room no. D3-226, Meibergdreef 9, 1105 AZ Amsterdam, North-Holland, the Netherlands; 2 Capgemini Invent, PO box 2575, 3500 GN Utrecht, Utrecht, the Netherlands; 3 Trauma Centre Southwest Netherlands, Erasmus Medical Centre, Dr. Molewaterplein 40, 3015 GD Rotterdam, South-Holland, the Netherlands; 4 National Health Care Institute, PO Box 320, 1110 AH Diemen, North-Holland, the Netherlands

**Keywords:** Urinary tract infections, Cystitis, Primary care, General practice, Shared decision making

## Abstract

**Background:**

Urinary tract infections (UTIs) are common, especially among women. Antibiotics are commonly used to treat UTIs, but might not always be necessary, for example in the case of uncomplicated UTIs such as cystitis. Shared decision making (SDM) could reduce the risk of unnecessary antibiotic prescriptions for uncomplicated cystitis. We investigated the current management and the use of SDM for uncomplicated cystitis in primary care.

**Methods:**

We performed a qualitative semi-structured interview study among 23 women with a history of cystitis, 12 general practitioner (GP) assistants, and 12 GPs in the Netherlands from July to October 2020. All interviews were individually performed by telephone. The data were analyzed through the use of using open and axial coding.

**Results:**

The GP assistants managed the initial diagnostics and treatment of uncomplicated cystitis in all general practices. Usually, antibiotics were considered the standard treatment of cystitis. In most general practices, SDM was not used in the treatment of uncomplicated cystitis, mainly because of a lack of time. Women reported that they valued being involved in the treatment decision-making process, but they were not always involved. Further, both GP assistants and GPs indicated that SDM would improve the care pathway of uncomplicated UTIs.

**Conclusion:**

In our study, SDM was infrequently used to help women with uncomplicated cystitis. To reduce the use of antibiotics for uncomplicated UTIs, a tailored intervention is needed to implement SDM for the treatment of uncomplicated cystitis in primary care.

## Background

Urinary tract infections (UTIs) are among the most common healthcare problems in primary care [1, 2]. Most UTIs are acute uncomplicated cystitis in women. The lifetime risk of cystitis for women is 60% and the risk of recurrence for UTIs in this group ranges from 30 to 50% per year [3]. Therefore, the burden of having (recurrent) cystitis is high and many women experience discomfort in their daily activities due to symptoms of cystitis or adverse events of antibiotic treatment [4–6].

Antibiotics are the most common treatment for UTIs. In fact, in 2018, most antibiotic prescriptions concerned UTIs in primary care in the Netherlands [1]. Since antibiotic resistance is increasing, non-antibiotic treatment options for uncomplicated cystitis have been researched and are increasingly supported in addition to antibiotic treatment [7, 8]. Therefore, guidelines for UTI, including the UTI guidelines for primary care in the Netherlands recommend, besides antibiotics, two other treatment options besides antibiotics [9–12]: (1) a wait-and-see policy with optional pain medication; and (2) a delayed antibiotic prescription, in which women receive an antibiotic prescription and have the opportunity to decide if they want to use antibiotics, based on their symptoms.

Remarkably, Knottnerus and colleagues already showed in 2013 that - when asked by their general practitioner (GP) - over a third of women with UTI symptoms are willing to delay antibiotic treatment [13]. Hence, women should be guided to make a well-informed decision about whether or not to use antibiotics for uncomplicated cystitis. This could be established through shared decision making (SDM); a process in which the pros and cons of different treatment options are discussed together with the patient, after which a decision is made together with the GP [14]. Results of previous studies showed that SDM could help women in choosing a tailored personalized treatment policy and reduce the use of antibiotics for uncomplicated cystitis [15, 16].

Although there is a growing number of initiatives that target the implementation of SDM in primary care in the Netherlands [17], it is currently unclear to what extent SDM is used in the treatment of uncomplicated cystitis. Besides, it is unknown whether women with uncomplicated or recurrent cystitis themselves would prefer the option of SDM when they contact their GP. We aimed to provide a better understanding of the management and use of SDM for uncomplicated cystitis in primary care in the Netherlands.

## Methods

### Study design and setting

A qualitative semi-structured interview study was performed from July to October 2020 among GPs, GP assistants, and women with a history of cystitis in the Netherlands. In the Netherlands, women suspected to have cystitis can visit the general practice and deliver a urine sample. The GP assistant is a medical assistant who is responsible for a variety of tasks (e.g., triage of patients, wound care, making appointments for patients) and who usually performs a urine dipstick test to diagnose or rule out a UTI. General practitioners in the Netherlands are physicians working in primary care who are also gatekeepers to hospital and specialist care. They could be compared to family physicians in the United States of America. We used the Consolidated Criteria for Reporting Qualitative Research (COREQ) checklist for comprehensive reporting [18]. This study was part of a bigger project: ‘Appropriate Care (Dutch: Zinnige Zorg)’ which was launched in 2013 by the National Health Care Institute in the Netherlands to improve patient care [19]. As part of this program, a project was set up to improve the care pathway for UTIs.

## Selection and recruitment of participants

To achieve heterogeneity in the sample of participants, purposive sampling was used in addition to convenience sampling. The GPs and GP assistants were mainly recruited through the personal network of a GP involved in the research team. Further, a leaflet with a summary of the study was spread among national scientific GP associations, social media groups, the national association of GP assistants, and the Public Health and Primary Care department of the Leiden University Medical Center.

Women with a history of cystitis were recruited through the participating general practices and through a leaflet with a summary of the study that was disseminated through social media groups. Women were eligible if they had visited their GP in the past year because of cystitis. For the distinction between types of cystitis, we used the definitions according to the clinical practice guideline for primary care in the Netherlands. Therefore, a recurrent cystitis was defined as having ≥ 3 cystitis per year [10].

All interested GPs, GP assistants, and women could apply for participation in this study by sending an e-mail to the research team. All participants received a voucher as compensation.

## Data collection and analysis

All interviews were based on interview guides and conducted by two senior researchers (GR and RvS). To gain as much adherence to daily practice as possible, the interview guides were conducted in close collaboration with a GP. The themes of the interview guide for GPs and GP assistants and women with cystitis are shown in Table 1. Further, we specifically asked the healthcare workers about the management of UTIs before the COVID-19 pandemic because this would give better insight into this care pathway. The sample size was based on data saturation and this was reached for GP assistants, GPs, and women with cystitis since we gained no new information on multiple themes in the last interviews.

**Table 1:** Interview guide

Topic	Subtopic	Group
UTI management	Role of GPs and GP assistants in management of UTI regarding diagnostics and treatment	GPs and GP assistants
	Which healthcare worker was contacted and in what way (e.g., face-to-face or by phone)?	Women
Counseling women with cystitis	What information is provided and what external information sources are used?	GPs and GP assistants
	How was the information transferred and how did women experience this?	Women
Shared decision making regarding UTI	To what extent is SDM used and is there a desire to use SDM?	GPs and GP assistants
	Desire to use SDM, why or why not?	Women
Implementation of SDM	From a woman’s perspective	Women

GP: general practitioner; SDM: shared decision making; UTI: urinary tract infection

All interviews were held in Dutch and, due to the COVID-19 pandemic restrictions, performed individually by telephone. We audio-recorded all but two interviews. The interview duration was 30 to 60 min. All recordings and notes were transcribed anonymously. The transcripts were coded and labeled through the use of open and axial coding [20]. Each code contained an explanation to promote uniform coding between the researchers. Codes were compared to each other and preliminary results were discussed by the researchers after they had coded the first interviews. Eventually, a consensus was reached over all codes. The preliminary results were discussed and interpreted with the research team, including a GP and an infectious diseases specialist. We used ATLAS.ti Windows desktop version 9.0 (Scientific Software Development GmbH, Berlin, 2022) for data analysis. The digital recordings, notes, and transcriptions were stored on a secured server. For the making of the appointments and sending of the vouchers, name, and address data were requested and stored secured and separately from the research data. After the research had finished, all source data, including digital recordings, were destroyed.

## Results

In total, 12 GPs, 12 GP assistants, and 23 women with (recurrent) cystitis were interviewed. These GPs and GP assistants worked in general practices located in both urban and rural areas in the Netherlands. The years of work experience of the GPs and GP assistants ranged from 3 to 35 years (median 12 years) and 1 to30 years (median 4 years), respectively. The ages of the participating women ranged from 25 to 79 years (median 48 years). The interviewed women lived in different areas in the Netherlands, both urban and rural, and their educational levels varied. One woman had experienced the first episode of cystitis, three women had experienced cystitis a few times in their lifetime, and the other women had experienced cystitis multiple times, varying from 1 to 5 times per year. Six of these women had recurrent cystitis in the past year. The main results that emerged from the interviews are listed in Table 2. These results are described in the following paragraphs.

**Table 2:** Main results

Topic	Result
UTI management in primary care	Substantial role for the GP assistant. Most consultations by phone. Referral to GP in certain cases (e.g., persisting symptoms, recurrent cystitis, signs of complicated UTI, and patients belonging to a risk group for complicated UTI).
Counseling women about UTI by healthcare workers	GP assistants provided information about preventive strategies (e.g., ample fluid intake). Women expected antibiotics to resolve their symptoms quickly. Most women found information about cystitis themselves.
Three treatment options and SDM	Few women were aware of the three treatment options for cystitis and the use of SDM. Both women and healthcare workers agreed that the use of SDM would improve the care pathway of UTIs.

GP: General practitioner; SDM: shared decision making; UTI: Urinary tract infection

## A substantial role for the GP assistant in the management of urine diagnostics and treatment of cystitis

In all general practices of the interviewed healthcare workers, GP assistants managed the initial urine diagnostics and treatment of uncomplicated cystitis in healthy non-pregnant women. Women with suspected cystitis contacted the general practice and then had a consult with the GP assistant (Woman 2). This consult was mostly by telephone. After this, the women answered questions about UTI-related symptoms and delivered a urine sample to the GP assistant. The GP assistants then followed the diagnostic recommendations from the national UTI guideline for primary care. All GPs and GP assistants reported that a urine dipstick test was always performed to diagnose cystitis and before antibiotics were prescribed as recommended in the UTI guideline for primary care (GP assistant 8). Antibiotic prescriptions for uncomplicated cystitis were drafted by the GP assistant, after which these were sent to the GP for approval. In most general practices, patients were only referred to their GP’s consulting hour in case of persisting symptoms during or after antibiotic treatment, recurrent cystitis, signs of complicated UTI, and when they belonged to a risk group (e.g., male gender, use of immunosuppressive medication, patients with multiple comorbidities) for complicated UTI (General practitioner 11).

*“Interviewer: And you delivered it (the urine sample) to the GP assistant?*

*Woman: Yes.*

*Interviewer: did you see your GP in those cases?*

*Woman: Actually, no. Now that you mention it, I received a phone call and usually a prescription for antibiotics which I could collect at the pharmacist (…)” (Woman 2)*.

*“In general, most women call our general practice and tell us their symptoms. For example, frequent urination or urge, sometimes fever or chills, or costovertebral pain. So, you try to report those symptoms as much as possible in the SOEP (patient’s medical record). And if possible, you want to check the urine.” (GP assistant 8)*.

*“Urinary tract infections are one of the most common symptoms that are mainly handled according to local protocols by our assistants. People do not visit the practice for that (…) They (assistants) report this (treatment) in the journal (medical record) and put it on a list for us to authorize.” (General practitioner 11)*.

## Women wished for their symptoms to be resolved quickly and therefore expected antibiotics

When women were diagnosed with cystitis, they received a phone call from their GP assistant during or after which antibiotics were prescribed (Woman 6). When asked for in the interview, some women were interested in non-antibiotic treatment options, but they were not provided with these options by their GP or the GP assistant. Therefore, they were unaware of these treatment options. Also, women with a history of recurrent cystitis wanted to consult their GP when they had a new episode of cystitis, even if this was not offered to them by the GP assistant. Further, most women with cystitis were affected by the symptoms in their daily activities and assumed that antibiotics would resolve their symptoms more quickly than the alternative -non-antibiotic- treatment options. Therefore, women were generally satisfied with their request for antibiotics when they had uncomplicated cystitis. Women who had already tried a wait-and-see policy themselves contacted the general practice in particular for an antibiotic prescription because of their persisting symptoms. Other important reasons for women to request antibiotics were the severity of their symptoms and the fear of developing a complicated UTI, such as pyelonephritis. Moreover, women preferred antibiotic treatment if they experienced severe symptoms or felt ill because of their cystitis, even though they were initially reluctant to use antibiotics because of side effects or fear of antibiotic resistance (Woman 14).

*“It genuinely goes like this: these are the results of your urine test, there are bacteria shown, and then an antibiotic prescription is sent to the pharmacist. As a patient, I do not have any influence on it.” (Woman 6)*.

*“I don’t really experience side effects or something, but I just know that it (use of antibiotics) is not good for your body. So I thought: I want to prevent that.” (Woman 14)*.

## Women valued being involved in the treatment decision-making process of uncomplicated cystitis

The majority of the interviewed women responded that no GP or GP assistant had informed them about the three treatment options for cystitis as recommended in the guideline for UTIs. Some women were partially aware of these treatment options as a result of their research or gained this information through their network of family and friends. Few women indicated that alternative treatment options besides an immediate antibiotic prescription were briefly discussed. A part of the interviewed women was not informed at all by their GP or GP assistant about the possible side effects or disadvantages of antibiotics. Further, most women mentioned that they valued being involved in the treatment decision-making process of cystitis, regardless of their wish for antibiotics. For example, a woman indicated that she would be open to trying a wait-and-see policy if her healthcare worker had explained that she could revisit the general practice if the symptoms did not resolve. Nevertheless, some women felt they had no influence at all on the initiated treatment (Woman 6) or were unaware of alternative treatment options for cystitis (Woman 1).

*“(…) Last time, I experienced that the symptoms could be resolved by myself. I just thought that this was impossible. The GP assistant never explained to me ‘Try to hold on for two days, make sure you drink lots of fluids and empty your bladder properly. Then it (cystitis) will likely cure itself.’ If someone had told me this before, then I would have tried this more often.” (Woman 1)*.

Only two women were fully included in an SDM process by their GP and a few women were partially familiar with SDM. However, most women were not involved in the treatment decision-making process at all concerning the treatment of their cystitis. Some women did not need SDM since they had already gained enough information about cystitis and its treatment or prophylactic strategies themselves. Apart from that, some women reported that they were aware that frequent use of antibiotics was harmful to their bodies (Woman 14), but they had not received this information from their GP. Further, especially women with recurrent cystitis reported that they would like to receive more information about prophylactic strategies (Woman 10).

*“They have never pointed it out or asked about it (prophylactic strategies). I noticed that I am affected by antibiotic treatment in some way (…) Therefore, I am curious for other options concerning treatment.” (Woman 10)*.

## Different motives for antibiotic prescriptions, including a presumed patient’s wish, limited time, and logistical barriers

The UTI guideline for primary care in the Netherlands recommended three treatment options for uncomplicated cystitis in healthy non-pregnant women. Nevertheless, GPs and GP assistants reported that antibiotics were the most commonly used treatment of cystitis. Some GPs assumed women expected antibiotics when they contacted the general practice and that women would not feel helped if a wait-and-see policy was proposed. Other GPs indicated that antibiotic treatment of cystitis was often carried out as a routine and half of them responded that the choice for antibiotic treatment was a standard procedure (GP 4 and GP 9). Further, GP assistants were more likely to agree with their patients’ requests for antibiotics if patients were severely affected by their symptoms (GP assistant 4). Besides, GP assistants reported that many patients who recognized their symptoms had already tried a wait-and-see policy (e.g., ample fluid intake, using cranberry juice or pills) before they contacted the GP general practice (GP assistant 7). Therefore, GP assistants did not recommend an additional wait-and-see policy to these patients.

In most general practices, the delayed prescription was only given to patients with recurrent cystitis who went on holidays or before the weekend started. Furthermore, logistical reasons and limited time were mentioned as important facilitators for directly prescribing antibiotics by both GPs and GP assistants. For example, GPs and GP assistants thought it would be invalidating and time-consuming for patients to visit the general practice twice when they had already visited once to deliver a urine sample. However, both GPs and GP assistants agreed that they would be more likely to discuss a wait-and-see policy with their patients if they had enough time to do so.

*“Yes it depends on the symptoms, you know. Look, if someone has such severe symptoms and does not want to wait for a week, because he or she wants her symptoms to be gone, then you just start treating as if it is cystitis.” (GP assistant 4)*.

*“Yes, and they recognized it (cystitis) and because they recognized their symptoms so clearly, they say ‘we tried to drink the symptoms away but our symptoms got worse or do not go away, so we would like antibiotics.’ And then we easily agree with them.” (GP assistant 7)*.

*“Well, I think that it is also a bit of a routine (laughs). Kind of, oh it’s cystitis, just treat it, you know. (…) I think it is some sort of convenient behavior, or how can you say it? A reflex-behavior (laughs).”(General practitioner 4)*.

*“My experience is that women always want an antibiotic cure because their symptoms are a true burden. So that’s mostly their request and in almost all cases, antibiotics are prescribed.” (General practitioner 9)*.

## Healthcare workers believed the use of shared decision making could improve the care pathway of uncomplicated cystitis

In most general practices of the interviewed healthcare workers, SDM was infrequently applied in the treatment of uncomplicated cystitis. Nevertheless, when GP assistants contacted their patients about confirmed cystitis, they explained to these patients that cystitis could be a self-limiting disease. The GP assistants also informed patients about additional non-antibiotic strategies (e.g., wait-and-see policy) and prophylactic strategies (e.g., ample fluid intake, proper emptying of the bladder). Regardless, the advantages and disadvantages of antibiotic treatment were often not discussed, which is also a part of SDM (GP assistant 5). In addition, only a few GPs and GP assistants reported that they referred their patients to information about cystitis that is available on GPinfo.nl (Dutch: Thuisarts.nl), a webpage conducted by the Dutch College of General Practitioners with information for patients [21]. Both GPs and GP assistants mentioned that it would benefit patients if they could spend time using SDM in the treatment of uncomplicated cystitis (GP assistant 6 and general practitioner 2). Next to this, GPs reported that they rarely applied SDM to the treatment of women with uncomplicated cystitis, since these women were mostly diagnosed and treated by the GP assistant. Therefore, some GPs stated that it would be useful to train GP assistants in SDM for these patients.

*“Interviewer: do you discuss the pros and cons of antibiotic treatment?*

*GP assistant: I think it sort of gets lost (…) I think that, not only concerning this (cystitis) that people, in general, are quite persistent in their wish for antibiotics.” (GP assistant 5)*.

*“ (…) In the end, it (providing patients with information) pays off, because we have people (patients in their general practice) who are nowadays saying that they tried a wait-and-see policy before contacting us (…) The amount of antibiotic use has decreased significantly, yes.” (GP assistant 6)*.

*“Lack of time makes it (use of shared decision making) difficult, in particular when patients have not thought about this or did not receive enough time to do so (…) I think a bit more explanation can save time in the long run, certainly in case of UTIs (…) Then people know that cystitis can resolve itself.” (General practitioner 2)*.

## Discussion

In general, SDM was mostly not and sometimes only partially used (i.e., by providing information about cystitis) for the treatment of uncomplicated cystitis. However, both women and healthcare workers agreed that the use of SDM would improve the care pathway for women with UTIs. Concerning the management of UTIs, GP assistants carried out diagnostics and drafted treatment proposals for uncomplicated cystitis in healthy non-pregnant women. This treatment mostly included antibiotics. In addition, some of the GPs still considered antibiotics the standard treatment of uncomplicated cystitis, and other GPs assumed that women with cystitis preferred antibiotics when they contacted their office. There were many different barriers to the use of SDM, including an assumption of a patient’s wish for antibiotics, lack of time, and logistical reasons. However, women mentioned that it would have benefited them if their GP had provided more information about the etiology, prophylactic strategies, and the different treatment options for uncomplicated UTIs. Further, only a few GPs and GP assistants referred women to informative websites, such as GPinfo.nl (Dutch: Thuisarts.nl).

In the past years, several studies focused mostly on GPs’ motives for following or deviating from clinical practice guidelines. Reported facilitators of prescribing antibiotics included limited time and patient factors, such as a patient’s expectation to receive antibiotics or if patients experienced severe symptoms of their disease [22–24]. We found similar facilitators of prescribing antibiotics in our study. In addition, we found that not only GPs but also GP assistants were more likely to propose antibiotic treatment to women with uncomplicated cystitis if their symptoms were a heavy burden. Also, women who were initially reluctant to use antibiotics because they feared side effects or adverse events, were nevertheless happy with antibiotics if they experienced severe symptoms of UTIs. Our results also suggest that women were insufficiently informed about the side effects of antibiotics and other treatment options that could relieve symptoms of cystitis, which is in accordance with the results of a previous qualitative interview study [25].

Although GPs’ assumptions that the use of SDM would take too much time and effort for women with cystitis, women were open to receiving information about non-antibiotic treatment options for uncomplicated cystitis if their GP took the time to counsel them. These findings are consistent with the results of previous studies [13, 26]. Further, the results of previous studies have shown that women with recurrent cystitis can recognize a new episode of cystitis properly [27, 28]. Women with recurrent cystitis could therefore benefit from sufficient self-management of uncomplicated cystitis. In our study, women appreciated it if their GP made an effort to investigate possible causes and discuss different treatment options, including prophylactic strategies, for cystitis. These findings are similar to those of other qualitative studies that investigated optimizing antibiotic stewardship regarding UTIs and the need for SDM in the treatment of UTIs [29–31]. It is therefore indicated that it is useful to provide women with proper consultations, in which healthcare workers listen to women’s experiences and discuss different treatment options with them. Further, most women with a history of cystitis in our study had gained information as a result of their experience with cystitis, which was also found in previously performed qualitative studies. The results of these studies showed a large variation in patient knowledge about UTIs and prophylactic strategies for recurring UTIs [30, 32]. Especially women in our study who had experienced pyelonephritis or other complicated UTIs indicated that they were afraid to develop this again when they had cystitis. However, the results of previous studies that compared the treatment of cystitis with antibiotics with placebo or pain medication showed that the risk of developing pyelonephritis was only slightly increased (incidence risk difference approximately 1.6%) when cystitis was not treated with antibiotics [33–36]. These findings indicate that proper counseling of women could be helpful in the treatment of UTIs.

A major strength of this study is that we interviewed women, GP assistants, and GPs about their roles in, and experience with the management of uncomplicated cystitis, whereas most previous studies focused on GPs or patients. Next, since women are most at risk of developing cystitis at least once in their lifetime, we believe we gained reliable information about the experience with the management of UTIs in primary care for healthy non-pregnant women. Further, we reached data saturation in all interviewed participant groups on all topics of interest in this study.

Our study also had limitations that should be taken into consideration. First, we could have a selection bias because of our recruitment strategy, as most participating general practices were recruited through the personal network of a GP in the research group. The participating general practices could be more interested in the subject because of this recruitment strategy. Nevertheless, we believe this bias is limited since the interviews were performed by two researchers who had no relationship with them. Moreover, the participating general practices, including their patients, did not belong to a UTI network. Second, most interviewed women had a history of recurrent cystitis. Women with the first episode of uncomplicated cystitis may have either more or less need for SDM in the treatment of uncomplicated UTI. Third, in qualitative research, questions could be asked about the generalizability of our results. In our study, this could be due to the sample size of included GP assistants, GPs, and women with a history of UTIs. However, we considered the number of GPs, GP assistants, and women in our study appropriate as we reached data saturation on all topics of our interview guides. Therefore, we believe we were able to provide an accurate insight into the current clinical practice in the Netherlands.

To reduce the number of antibiotic prescriptions for uncomplicated cystitis, women should be more informed about alternative treatment options. We found that women had limited experience with a delayed antibiotic prescription for uncomplicated cystitis, but valued being involved in the treatment decision-making process. Therefore, it could be helpful to spend more time counseling women about the different treatment options for uncomplicated cystitis. Together with this counseling, it might be valuable to conduct a proper medical history consultation about women’s symptoms, because a woman may have symptoms that could be attributed to other diseases than cystitis, e.g., sexually transmittable infections. Further, since the majority of women being interviewed in our study had a history of recurrent cystitis, they had become aware of strategies, such as proper emptying of the bladder, as a result of their own experiences. However, these findings suggest that women could be provided with information about treatment options for cystitis sooner.

Future research should also focus on barriers and facilitators of using SDM for the treatment of uncomplicated cystitis in primary care. Hence, a tailored intervention can be conducted to implement SDM for UTIs in primary care. Moreover, since GP assistants are in charge of the initial diagnostics and treatment of uncomplicated cystitis in healthy non-pregnant women, they should be involved in the implementation process of SDM. Based on the results of this study, we are now starting a follow-up study to investigate the barriers to implementing SDM for UTIs in primary care.

## Conclusion

In summary, SDM was infrequently used in the treatment of uncomplicated cystitis by the GPs and GP assistants. This was mostly due to a lack of time, the presumed patient’s wish for antibiotics, and because antibiotics were still considered the standard treatment option for cystitis. Women indicated feeling satisfied with antibiotic treatment of cystitis but also indicated that they would have appreciated the use of SDM. To reduce the risk of unnecessary antibiotic treatment, the use of SDM in primary care in the care pathway of UTIs should be improved.

## Data Availability

The datasets used and/or analysed during the current study are available from the corresponding author on reasonable request.

## List of abbreviations

COREQ: Consolidated Criteria for Reporting Qualitative Research.
GP: general practitioner.
SDM: shared decision making.
UTI: urinary tract infection.
